# The Process of Labour

**Published:** 1844-08-24

**Authors:** 


THE PROCESS OF LABOUR.

Mr. Tyler Smith says, it may be stated briefly that labour consists of positive dilatation of the os uteri and the vagina, the action of the muscles of expiration, and contraction of the vagina; all excito-motory phenomena, aided by volition, and modified by emotion.—*Ibid.*

